# Data-Driven Adaptive Tracking Control for Nonlinear New Quality Productive Forces Systems with Input Constraints

**DOI:** 10.3390/e28060598

**Published:** 2026-05-27

**Authors:** Siao Liu, Yongjiu Li, Chunxiao Sun, Yi Wang, Shuxian Ji

**Affiliations:** 1 School of Economics, Zhejiang University of Science and Technology, Hangzhou 310023, China; liusiao8877@163.com (S.L.); 17354183518@163.com (Y.L.); 113009@zust.edu.cn (S.J.); 2 College of Zhijiang, University of Zhejiang Science and Technology, Shaoxing 312030, China; 3 School of Data Sciences, Zhejiang University of Finance & Economics, Hangzhou 310018, China; wangyishuke@zufe.edu.cn

**Keywords:** data-driven control, adaptive control, system identification, constrained optimization, new quality productive forces, 93C40

## Abstract

This paper addresses issues such as nonlinearity, model uncertainty, and multiple policy constraints within the dynamic evolution of new quality productive forces systems. It proposes a research framework integrating data-driven modelling with adaptive tracking control. By merging control theory with economic dynamics, a closed-loop analytical system of ‘theory-data-control’ is constructed, providing a methodologically rigorous yet operationally feasible pathway for the precise regulation of complex economic systems. First, utilising provincial panel data, a discrete-time system model integrating linear inertia, policy effects, and nonlinear compensation is established. System parameter identification is achieved through a dual machine learning approach employing partial linear regression. Subsequently, a tracking controller integrating data-driven initial identification with online parameter adaptation is designed, incorporating a projection mechanism to strictly ensure policy variables remain within feasible adjustment ranges. Based on Lyapunov stability theory, we demonstrate that the tracking error of the closed-loop system exhibits ultimate convergence with boundedness. Simulation experiments confirm that the proposed method significantly enhances the system’s tracking performance towards the target trajectory, reducing the mean absolute error by approximately 30.8% while producing smoother control signals. Comparative studies indicate that the parameter adaptation mechanism and nonlinear compensation module play crucial roles in improving control effectiveness. This research not only expands the theoretical toolkit for analysing the dynamics of new quality productive forces but also provides an interdisciplinary methodological reference for the closed-loop management of complex socioeconomic systems under data-driven conditions.

## 1. Introduction

The core of adaptive control lies in maintaining the system’s expected performance and stability under parameter uncertainties and external disturbances. In recent years, the integration of data-driven methods with classical adaptive frameworks has offered new insights: machine learning can extract system features from data to assist controller design, while adaptive laws based on Lyapunov stability adjust parameters online to cope with dynamic changes. This “data-driven identification + adaptive control” paradigm has demonstrated potential in mechatronic systems [1], but its application in socioeconomic systems characterized by strong nonlinearity, multiple uncertainties, and hard constraints remains limited. Traditional economic methodologies often rely on linear or linearizable models, struggling to adequately capture complex dynamics like the evolution of “new quality productive forces”, which involve multi-factor nonlinear interactions, structural time-varying properties, and constraints. Therefore, introducing systematic nonlinear adaptive control theory into this field to construct an analytical framework combining explanatory power and regulatory capability emerges as a promising research direction. Against this backdrop, this paper addresses the dynamic regulation of new quality productive forces systems by investigating data-driven identification of their discrete-time nonlinear models and developing adaptive tracking control methods under constraints. The objective is to provide a theoretically rigorous and practically feasible control framework for closed-loop regulation of complex economic systems.

Traditional research on new quality productive forces has primarily focused on its economic implications, measurement, and macroeconomic effects. On one hand, studies examining factor upgrading perspectives highlight its manifestation as comprehensive qualitative transformations in laborers [2], means of labor [3], and objects of labor [4]. On the other hand, from the perspective of new combinations, it emphasizes the new integration of factors driven by technological innovation [5]. While these studies profoundly reveal its essence, they are mostly conducted at the static or macro-statistical level.

When examined through the lens of dynamic systems and control theory, the entities underpinning new quality productive forces (intelligent manufacturing, digital finance) constitute a quintessential complex nonlinear dynamic system. Its “new quality” manifests as high-order dynamics, strong coupling, and time-varying parameters within the system; its “integration” corresponds to high-dimensional hybridity in system states (interweaving continuous dynamics and discrete events). These systemic characteristics pose challenges that traditional model-driven control methods struggle to address: First, precise mechanistic models are difficult to obtain, with significant “unmodeled dynamics” present. Second, system operation is subject to strict physical or safety constraints (state limitations, input saturation). Third, the control system must achieve precise tracking and regulation performance even under high uncertainty. For instance, in safety-critical cyber–physical systems, unknown deception attacks on sensor and actuator networks can severely degrade performance, necessitating resilient adaptive schemes to maintain stability and tracking accuracy under such adversarial conditions [6]. Although existing research has examined macroeconomic effects [7,8], few studies have systematically analysed the underlying control theory issues from a systems dynamics and cybernetics perspective. This constitutes the entry point for our research question.

To address these challenges, nonlinear adaptive control theory offers a powerful toolkit. Among these, the backstepping method, as a systematic design framework, demonstrates significant advantages in solving high-order nonlinear system control problems, with its modular design facilitating the handling of uncertainties [9]. This methodology has been progressively refined and enhanced in practice. When integrated with finite-time stability theory, it enables the design of high-performance trajectory tracking controllers for quadcopter drones operating under model uncertainty and external disturbances [10]. Concurrently, its framework has been successfully applied to the precise control of uncertain robotic manipulators, effectively resolving distinct design challenges arising from state and output feedback configurations [11]. For constrained problems where system states or outputs must operate within safe regions, the barrier Lyapunov function approach has emerged as a mainstream solution. This approach constructs Lyapunov functions that tend to infinity at the constraint boundaries, thereby a priori ensuring that state constraints are not violated. Ref. [12] applied it to pure feedback systems, Ref. [13] to uncertain nonlinear systems, and Ref. [14] successfully addressed state-constrained control for quadcopter drones, demonstrating its engineering viability. Yet achieving rapid convergence within user-specified time bounds while enforcing state constraints remains challenging. Ref. [15] addressed this via a predefined-time framework that combines barrier Lyapunov functions with command-filtered backstepping, guaranteeing deadline-based convergence under state constraints while maintaining high tracking accuracy and reducing proof complexity.

Furthermore, to simultaneously optimize system performance under constraints, researchers integrated control Lyapunov functions with barrier functions into constrained optimization control frameworks. Adaptive robust optimization and quadratic programming approaches proposed by Refs. [16,17] represent this direction with their adaptive robust optimization and quadratic programming methods, respectively, enabling online determination of optimal control laws satisfying multiple constraints and stability conditions. This framework is being integrated with data-driven paradigms. For instance, data-driven adaptive methods based on immersion and invariance principles have been employed to address the dual-level state-constrained control problem for rigid vehicles [18]. Concurrently, data-driven event-triggered optimal control frameworks have demonstrated their potential for fully unknown nonlinear systems with constrained inputs [19]. However, traditional adaptive control heavily relies on structural information of the system model (nonlinear parameterized form). When confronting more complex “unmodeled dynamics” and high-dimensional mixed factors in new quality productive forces systems, this model dependency becomes a major bottleneck. Even when introducing event-triggered mechanisms within traditional adaptive frameworks to address resource constraints [20] or augmenting such controllers with neural network estimators to compensate for unmodeled dynamics [21], their core remains constrained by prior assumptions regarding the system model structure. When confronted with entirely novel or highly uncertain dynamics, a fundamental shift towards data-driven learning and compensation strategies becomes particularly imperative.

Data-driven control methods offer a novel approach to overcoming these limitations. Such methods do not rely on precise mechanistic models but instead utilize system operational data to directly or indirectly design controllers, demonstrating particular proficiency in handling complex nonlinearities and unmodeled dynamics. Current research exhibits a strong convergence trend: on one hand, adaptive control provides a theoretical framework for stability assurance in data-driven methods [22]; on the other, data-driven approaches significantly enhance adaptive controllers’ learning and compensation capabilities for complex unknown dynamics. For instance, Ref. [23] reviewed data-driven control methods based on semidefinite programming, demonstrating their stability design under model-free conditions. Refs. [24,25] advanced the application of data-driven adaptive control in practical nonlinear systems by focusing on optimizing data utilization and model-free predictive control, respectively. This advancement has achieved significant progress in complex systems [26] demonstrating its potential for addressing large-scale, strongly nonlinear practical problems. At the algorithmic theory level, the foundational framework for data-driven predictive control of nonlinear systems has also undergone continuous refinement to enhance its consistency and computational efficiency [27]. Furthermore, approaches based on meta-learning [28] and adaptive dynamic programming [29] have further enhanced control systems’ ability to rapidly learn from data and approximate optimal performance.

This paper addresses the data-driven modeling and adaptive tracking control problem for the complex economic dynamic system of new quality productive forces evolution, characterized by typical nonlinearity, uncertainty, and multiple constraints. The main contributions are as follows:

1. An integrated theory-data-control analytical framework is constructed, introducing nonlinear system control theory into new quality productive forces research and achieving a closed-loop from data-driven modeling to constrained optimization control. 2. Developed a system model combining interpretability and flexibility. An affine discrete-time representation captures linear inertia, policy effects, and complex nonlinear dynamics. 3. Designed a control strategy integrating data-driven initial identification with online adaptive tracking. Employed the PLR-DML method for robust parameter estimation and designed an adaptive controller incorporating projection operations to rigorously handle multi-policy constraints. 4. A closed-loop analysis was completed, spanning rigorous theoretical proofs to comprehensive simulation validation. The stability and consistent ultimate boundedness of the closed-loop system were demonstrated using the Lyapunov method. The framework’s effectiveness and robustness were verified through numerical simulations based on provincial panel data.

## 2. System Modelling

This paper considers a nonlinear uncertain discrete-time dynamic system. For any province, the state variable at discrete time t=0,1,2,… is denoted as $x_{t}\in R$, representing the province’s new quality productive forces composite index at time t. Regarding the measurement of new quality productive forces, this paper adopts the indicator system approach and employs the entropy weighting method within the objective weighting approach to assign weights to indicators. Currently, scholars have not reached consensus on constructing an indicator system for measuring new quality productive forces. Most scholars build frameworks based on dimensions such as new quality laborers, new quality means of labor, and new quality objects of labor, with specific indicators varying across dimensions. Drawing upon Marx’s theory of productive forces and referencing the research of [30,31], this paper adheres to the aforementioned principles in selecting specific indicators to construct an indicator system for new quality productive forces. This framework comprises three primary indicators: new quality laborers, new quality labor objects, and new quality labor resources, as detailed in Table 1. The controllable policy variables adopted in this study are: the level of fiscal subsidies for scientific and technological innovation (fiscal expenditure on scientific and technological innovation/total government fiscal expenditure), investment in social welfare security (fiscal expenditure on social security and employment/total government fiscal expenditure), and the level of digital finance [32]. Data are sourced from the National Bureau of Statistics of China (2012–2023).

Clarification of the data structure and identification strategy: the empirical sample is a balanced provincial panel indexed by province and year over 2012–2023. In the baseline identification, the structural parameters are estimated from the pooled provincial panel rather than separately by province, because the annual time dimension for each province is short. Province fixed effects and year effects are included and partialled out in the PLR-DML nuisance stage to absorb time-invariant regional heterogeneity and common macro shocks. The controller is then rolled out province by province using each province’s observed initial state and policy inputs.

To clarify the variable definitions, component meanings, bounds, and projection intervals used in the subsequent identification and control design, the key variables are summarised in Table 2.

To facilitate controller design, all provinces are regarded as parallel subsystems with the same structural form. The following derivation employs a representative single-province system as an exemplar, while the empirical rollout is implemented province by province.

The system’s control input is a three-dimensional vector comprising three controllable policy factors: digital financial development level ($DF_{t}$), level of fiscal subsidies for technological innovation (${SUB}_{t}$) and social security expenditure (${SOC}_{t}$). These three policy variables are combined into

(1) $$\;u_{t}={\left[DF_{t},\;{SUB}_{t},\;{SOC}_{t}\right]}^{T}\in R^{3}$$

Three types of policy variables jointly influence the evolution of new quality productive forces. Given the pronounced nonlinearity and structural uncertainty within the macroeconomic system, this paper models system dynamics as nonlinear state equations with affine inputs:

(2) $$x_{t+1}=ax_{t}+b^{T}u_{t}+\phi {\left(x_{t}\right)}^{T}\theta +w_{t}$$

(3) $$\;\;b^{T}u_{t}=b_{1}{DF_{t}+b}_{2}{SUB}_{t}+b_{3}{SOC}_{t}$$

Here, a∈R represents the linear “inertia” coefficient of the state, characterising the autoregressive effect of new quality productive forces, *b =* ${\left[b_{1},b_{2},b_{3}\right]}^{T}\in R^{3}$ denotes the linear gain vector corresponding to each channel of the control input, $\phi (x_{t})\;\in R^{m}$ denotes the nonlinear basis function regarding the state, with its parameter vector $\theta \in R^{m}$, and $w_{t}$ represents the error caused by unmodelled dynamics and external disturbances. Equation (2) approximates changes in new quality productive forces levels by decomposing them into linear and nonlinear components: $ax_{t}$ represents the intrinsic growth or decline trend of new quality productive forces, $b^{T}u_{t}$ quantifies the direct linear marginal impact of three policy inputs on productivity; $\phi {\left(x_{t}\right)}^{T}\theta$ compensates for nonlinear effects, time-varying factors, or structural biases not captured by the linear component. Given the inherent complexity of the objective system, coefficients a, *b* and *θ* are treated as unknown and uncertain, they may vary over time or introduce bias due to model simplification, while $\epsilon_{t+1}$ represents random disturbances that cannot be precisely described. Compared to traditional purely linear models, this framework incorporates $\phi (x_{t})$ to capture dynamics beyond linear explanation, yielding a more realistic system characterization. Separating linear parameters a and b preserves interpretability regarding policy marginal effects and inertia effects. For subsequent control design convenience, denote the system parameter vector as $\vartheta ≜{[\;a\;b^{T}\;\theta^{T}]\;}^{T}$.

For readability, the model can be interpreted as the sum of four parts: the inertia part, the direct policy-input part, the nonlinear compensation part, and the disturbance part. The inertia part captures persistence in the new quality productive forces index; the policy-input part measures the marginal contribution of digital finance, innovation subsidy intensity, and social security expenditure; the nonlinear compensation part captures dynamics not well represented by the linear component; and the disturbance part collects unmodelled shocks.

The control objective of this paper is to design an appropriate sequence of policy inputs $\left\{u_{t}\right\},$ such that the system state $x_{t}$ can track a predefined reference trajectory $x_{t}^{ref}$. The reference trajectory is assumed to be a known, smooth, and bounded sequence, corresponding to the target path for new quality productive forces established under economic development planning. To measure tracking performance, the tracking error is defined as:

(4) $$\;\;\;\;e_{t}=x_{t}-x_{t}^{ref}$$

When $e_{t}=0$, it indicates that the new quality productive forces have precisely met the planned targets; When $\left|e_{t}\right|$ is relatively large, it signifies that the level of new quality productive forces in that province has deviated significantly from expectations.

The control objective pursued in this paper may be formulated as follows: to devise a feedback policy control law $u_{t}=k_{t}(\cdot )$ based on observable states and historical information, such that the closed-loop system, whilst satisfying policy constraints, either causes the tracking error $e_{t}$ to asymptotically approach zero, or maintains it within a bound interval that is as small as possible in the presence of external disturbances.

Considering the constraints and physical limitations during policy implementation, the following fundamental assumptions are made regarding the system:

Assumption 1: Observability and Information Structure. At each time step t, the decision-maker can observe the current state $x_{t}$, the historical inputs ${{\{u}_{T}\}}_{T\leq t-1}$, and observable exogenous variables relevant to the system. The reference trajectory $\;x_{t}^{ref}$ is pre-specified and known throughout the entire control interval.

Assumption 2: Input Constraints. Policy inputs $u_{t}$ must belong to a specified feasible set.

$$U=\left\{u\in R^{3}:u_{min}\leq u\leq u_{max}\right\}$$

The inequality should be interpreted component-wise, with $u_{min}$ and $u_{max}$ determined by the upper and lower bounds of the actual policy. The feasible set U is non-empty, closed, and bounded.

In the empirical implementation, the admissible input set is determined by the observed feasible ranges of the three policy variables. In raw units, the digital finance input is restricted to [61.47, 498.28], the innovation-subsidy input to [0.004785, 0.067569], and the social-security input to [0.078238, 0.299718]. Since the simulation standardises the control variables before the rollout, the corresponding projection intervals used in the numerical controller are [−2.2091, 2.3669] for digital finance, [−1.1343, 2.8890] for innovation subsidy, and [−1.6566, 4.1692] for social security expenditure.

Assumption 3: Boundedness of nonlinear terms and perturbations. The nonlinear basis function ϕ(·) is continuous and Lipschitz continuous within the considered state domain, i.e., there exists a constant $L_{\phi }>0$ such that

(5) $$\;\left\|\phi \left(x_{1}\right)-\phi \left(x_{2}\right)\right\|\leq L_{\phi }\left|x_{1}-x_{2}\right|,\;\;\forall x_{1},x_{2}$$

External perturbations $\epsilon_{t}$ are bounded, that is, there exists a constant $\bar{\epsilon_{t}}>0$ such that $\left|\epsilon_{t}\right|\leq \bar{\epsilon_{t}},\forall t$.

The aforementioned assumptions form the basis for subsequent stability analysis: Assumption 1 guarantees the realisability of the error feedback control law; Assumption 2 ensures the control input satisfies practical policy constraints; Assumption 3 prevents the system’s nonlinear component from ‘exploding’, thereby facilitating the construction of a Lyapunov function and proving the uniform boundedness of the closed-loop error.

Prior to the actual design of the controller, model parameters must be identified using historical data. Given that the model incorporates both linear and nonlinear terms, this paper employs Partially Linear Regression Double Machine Learning (PLR-DML).

Perform data-driven identification of parameters (a,b,θ). This method combines the flexible fitting capabilities of machine learning with the interpretability of traditional regression, enabling robust consistent estimation even in the presence of high-dimensional control variables or complex nonlinear confounding factors.

Assume an existing system time series $\;\{x_{t},u_{t},Z_{t}\},$ where $Z_{t}$ denotes other observable variables potentially influencing the evolution of new quality productive forces beyond state and control inputs (economic structure, industrial structure, demographic structure, etc.). Defining the dependent variable as $Y_{t}=x_{t+1}$ and the treatment variable as ${D_{t}=(x_{t},u_{t},\phi \left(x_{t}\right))}^{T}$, the original system model can be expressed as the following partial linear form:

(6) $$Y_{t}=D_{t}^{T}\vartheta +g(\left(Z_{t}\right))+\varepsilon_{t}$$

Here, ϑ denotes the structural parameter to be estimated, $g(Z_{t})$ represents an unknown nonlinear function of control variables, and $\varepsilon_{t}$ constitutes the noise term. Ideally, if $g(Z_{t})$ were known, ϑ could be directly estimated via regression. However, in real-world macroeconomic scenarios, $g(Z_{t})$ is complex and high-dimensional, rendering its prior parameterisation challenging. The core concept of PLR-DML is to employ machine learning methods to flexibly estimate the conditional expectations of $g(Z_{t})$ and $D_{t}$, followed by traditional linear regression in the residual space to obtain a robust and consistent estimate of ϑ.

PLR-DML achieves parameter estimation through the following two stages:

Employing appropriate machine learning methods, fit separately on the entire sample or on a training subset:

(7) $$\;{\hat{m}}_{Y}\left(Z_{t}\right)\approx E\left[Y_{t}|Z_{t}\right],\;\;{\hat{m}}_{D}\left(Z_{t}\right)\approx E\left[D_{t}|Z_{t}\right]$$

Calculate the residuals of the results and the treatment residuals on the validation subsample:

(8) $$\;\;{\tilde{Y}}_{t}=Y_{t}-{\hat{m}}_{Y}\left(Z_{t}\right),\;\;\;\;\;\;\;\;\;{\tilde{D}}_{t}=D_{t}-{\hat{m}}_{D}\left(Z_{t}\right)$$

This step serves to orthogonalise the linear regression vector with respect to correlations among high-dimensional confounding variables, such that ${\tilde{Y}}_{t}$ and ${\tilde{D}}_{t}\;$ become conditionally approximately orthogonal to $Z_{t}$. This thereby mitigates the endogeneity bias introduced by high-dimensional confounding.

To mitigate the risk of overfitting in the first-stage machine learning model, this paper employs cross-validation: dividing the samples into K folds, training ${\hat{m}}_{Y}$ and ${\hat{m}}_{D}$ using K-1 folds of data each time, and calculating residuals (8) on the remaining 1 fold. After iterating through all folds, the residuals are aggregated. This strategy significantly reduces the interference of machine learning errors on the second-stage linear regression.

Implementation details for reproducibility are as follows. The PLR-DML procedure uses K = 5 cross-fitting folds. The nuisance functions are estimated by Random Forest regressors with the same fold partition; LASSO regression is used as a sensitivity learner to check whether the estimated signs and relative magnitudes of the structural parameters are robust. Continuous variables are standardised before estimation. Province and year indicators are included in the nuisance covariates so that fixed provincial heterogeneity and common year shocks are absorbed before the second-stage orthogonal regression. Standard errors are computed from the residualised second-stage regression and clustered at the province level.

Upon obtaining the residual sample ${\left\{{\tilde{Y}}_{t},{\tilde{D}}_{t}\right\}}_{t=0}^{T-1}$, perform ordinary least squares regression:

(9) $$\;\;\;\hat{\vartheta }=arg{min}_{\vartheta }\sum_{t=0}^{T-1}{\left({\tilde{Y}}_{t}-{\tilde{D}}_{t}^{⊺}\vartheta \right)}^{2}$$

Under appropriate regularity conditions, the aforementioned estimator $\hat{\vartheta }$ exhibits asymptotic normality. Its covariance matrix $\hat{Var}(\hat{\vartheta })$ and corresponding confidence intervals may be obtained through standard linear regression theory:

(10) $$\hat{\vartheta }\pm z_{1-\frac{\alpha }{2}}\sqrt{diag(\hat{Var}(\hat{\vartheta })}$$

In this paper, particular attention is paid to the structural parameters $\hat{a}$, $\hat{b}$ and $\hat{\theta }$, which are employed as the initial parameters for the controller in Section 3:

$${\hat{a}}_{0}=\hat{a},\;\;\;\;{\hat{b}}_{0}=\hat{b},\;\;\;\;{\hat{\theta }}_{0}=\hat{\theta }$$

To enhance stability during the initial phase of the closed-loop system, parameter handling may be adjusted conservatively in conjunction with confidence intervals, with the modified initial values serving as the starting point for the adaptive control law.

Through the aforementioned PLR-DML identification procedure, this paper retains the economic significance and interpretability of the structural parameters in Model (1) while fully leveraging the fitting capabilities of machine learning in high-dimensional, nonlinear environments. This provides a reliable parameter foundation for subsequent model-based adaptive control design and stability analysis.

## 3. Controller Design

Following the establishment of the model and estimation of initial parameters, this paper designs a multi-input multi-output (MIMO) trajectory tracking controller based on tracking error feedback and online parameter adaptation. A channel-independent update strategy is adopted. The error dynamics equation for the closed-loop system is derived below, from which the control law structure is formulated. The tracking error is defined as $e_{t+1}=x_{t+1}-x_{t+1}^{ref}$. Integrating with the model, the error dynamics can be derived and reorganised as:

(11) $$\;e_{t+1}=\left(a-{\hat{a}}_{t}\right)x_{t}+{\left(b-{\hat{b}}_{t}\right)}^{T}u_{t}+{\left(\theta -{\hat{\theta }}_{t}\right)}^{T}\phi \left(x_{t}\right)-Ke_{t}+\varepsilon_{t+1}$$

Here, K>0 denotes the feedback gain, employed to enhance error convergence characteristics. This equation indicates that error evolution is jointly influenced by parameter estimation error, active error correction $\left(Ke_{t}\right)$, and external disturbance $\left(\varepsilon_{t+1}\right)$. To enable the system state to track the reference trajectory, the following control law is proposed:

(12) $$u_{t}=Proj\{\frac{x_{t+1}^{ref}-{\hat{a}}_{t}x_{t}-{\hat{\theta }}_{t}^{T}\phi \left(x_{t}\right)+Ke_{t}}{{\hat{b}}_{t}}\}$$

The fractional term denotes the difference between the reference trajectory at the next time step and the model prediction output, which is scaled and distributed by the estimated input gain ${\hat{b}}_{t}$ after correction by the feedback gain; Proj{⋅} represents the appropriate projection processing applied to the resulting ideal continuous control quantity to ensure each control component remains within a reasonable range. Intuitively, the control law endeavours to approximate the predicted $x_{t+1}$ to $x_{t+1}^{ref}$ at each step. Upon selecting an appropriate K, this control scheme not only compensates for known model errors, but also compensates for the system’s inherent dynamics and nonlinear effects through $\left\{-{\hat{a}}_{t}x_{t}-{\hat{\theta }}_{t}^{T}\phi \left(x_{t}\right)\right\}$, directing the control input primarily towards overcoming residual discrepancies. This design strategy combines feedforward compensation with feedback correction.

As the model parameters are not precisely known, this paper introduces an adaptive parameter update rule to adjust the parameter estimates $\left({\hat{a}}_{t},{\hat{b}}_{t},{\hat{\theta }}_{t}\right)$ online during system operation, thereby progressively approximating the true parameters. The parameter update rule is designed based on the predictive error correction principle: when tracking errors occur, the direction of parameter adjustment is modified to minimise the error in the subsequent step.

Given that all parameters in the model exhibit linear uncertainty, this paper employs a gradient-type adaptive law, whose general form is defined as ‘the product of the parameter error and the corresponding regression signal’. The specific discrete-time update formula is designed as follows:

(13) $$\;\;\;{\hat{a}}_{t+1}={\hat{a}}_{t}-\gamma_{a}\cdot x_{t}\cdot e_{t}$$

(14) $$\;\;{\hat{b}}_{k,t+1}={\hat{b}}_{k,t}-\gamma_{b_{k}}\cdot u_{k,t}\cdot e_{t}$$

(15) $$\;{\hat{\theta }}_{t+1}={\hat{\theta }}_{t}-\gamma_{\theta }\cdot \phi \left(x_{t}\right)\cdot e_{t}$$

Here, $k=1,2,3,\;\gamma_{a},\gamma_{b_{k}},\gamma_{\theta }>0$ denote the gradient gain coefficients of the adaptive law, while $u_{k,t}$ represents the control input signals for the three channels. The aforementioned update law continuously refines parameter estimates to minimise the impact of model uncertainty on closed-loop performance. In practical applications, projected processing is applied to the parameter estimates, constraining their update range within a reasonable historical experience interval to prevent undesirable outcomes such as divergence or sign errors in the estimates. This projection operation can be unified with the projection in the control law, ensuring the overall robustness of the controller.

In practical applications, control inputs and parameter estimation must adhere to physical and strategic constraints. Consequently, appropriate limiting mechanisms must be incorporated into the implementation of the aforementioned ideal control laws and adaptive laws. Projection saturation constraints, denoted as $Proj\left\{\cdot \right\}$, represent a component-wise projection saturation operation. When computed control input candidates exceed historical feasible ranges or physically permissible bounds, they are truncated within preset intervals. Projection constraints are also applied to parameter estimates $\left({\hat{a}}_{t},{\hat{b}}_{t},{\hat{\theta }}_{t}\right)$ to ensure they remain within reasonable ranges. Specifically, it is essential to prevent the estimated gain vector ${\hat{b}}_{t}$ from approaching zero excessively or undergoing sign reversal, as ${\hat{b}}_{t}$ occupies the denominator position in the control law. Both scenarios introduce singularity and stability hazards into the control computation.

Operationally, the controller first computes an unconstrained candidate input, denoted as ${\tilde{u}}_{t}$ and then applies a component-wise projection before implementation:

(16) $${\left[{Proj}_{U}\left({\tilde{u}}_{t}\right)\right]}_{k}=\min\left\{u_{k,max},max\left\{u_{k,min},{\tilde{u}}_{k,t}\right\}\right\},k=1,2,3.$$

Thus, if the feedback law generates a policy input outside the admissible range, the implemented input is saturated at the corresponding lower or upper bound. This is how the input constraints are incorporated directly into the control design.

Adaptive gain adjustment: The gain coefficients $\left(\gamma_{a},\gamma_{b_{k}},\gamma_{\theta }\right)$ within the gradient adaptive law directly influence the convergence speed and stability of parameters. Excessively high gain values may cause severe oscillations in parameter estimation and error during updates, while excessively low values result in slow convergence. Consequently, these gains are adjusted based on empirical evidence or through simulation, with the magnitude of error and parameter changes monitored during operation. Furthermore, as the influence of each input channel on the system varies, distinct initial values are assigned to $\gamma_{a},\gamma_{b_{k}}$ and $\gamma_{\theta }$ to reflect the sensitivity of each channel.

For the comparison experiments, three controller variants are defined consistently with the control design. The Full Controller uses the projected feedback control law, the online adaptive updates, and the nonlinear compensation term. The Fixed-parameter Controller keeps the projected feedback law but disables the online updates, using only the initial PLR-DML estimates. The No nonlinear compensation controller removes the nonlinear compensation term while retaining the projected feedback and parameter update mechanisms for the linear part. These definitions isolate the roles of online parameter adaptation and nonlinear compensation.

## 4. Stability Analysis

To analyse the stability of the closed-loop system, this paper employs the second Lyapunov method. First, a candidate positive definite Lyapunov function is constructed for the system. Based on the aforementioned error dynamics and parameter update form, a quadratic function incorporating both the tracking error and the squared parameter estimation error is selected:

(17) $$\;V_{t}=\frac{1}{2}e_{t}^{2}+\frac{1}{2\gamma_{a}}{\left(a-{\hat{a}}_{t}\right)}^{2}+\sum_{k=1}^{3}\frac{1}{2\gamma_{b_{k}}}{\left(b_{k}-{\hat{b}}_{k,t}\right)}^{2}+\frac{1}{2\gamma_{\theta }}{\left\|\left.\theta -{\hat{\theta }}_{t}\right\|\right.\;}^{2}$$

where $e_{t}$ denotes the tracking error, $\left(a-{\hat{a}}_{t}\right)$, $\left(b_{k}-{\hat{b}}_{k,t}\right)$, and $\left(\theta -{\hat{\theta }}_{t}\right)$ represent the estimation errors for parameters a, $b_{k}$, and θ respectively, while $\gamma_{a},\gamma_{b_{k}}$ and $\gamma_{\theta }$ denote the positive gradient gains corresponding to the adaptive law.

In the Lyapunov function, the scalar coefficient associated with the nonlinear-parameter error multiplies the entire squared norm of that error term; it is not included inside the brackets of the parameter error expression.

According to the above construction, $V_{t}\geq 0$ holds for all $e_{t}$ and parameter errors, and $V_{t}=0$ occurs if and only when $e_{t}=0$ and all parameter estimation errors are zero. Therefore, $V_{t}$ is a positive definite function with respect to the error state. When the closed-loop system operates near the origin equilibrium point of tracking errors and parameter estimation errors, this Lyapunov function is expected to decrease gradually, thereby ensuring system stability.

Next, we examine the change in the Lyapunov function between adjacent discrete time steps, namely deriving $V_{t+1}-V_{t}$. Utilising the error dynamics equation and the parameter update rule, we expand $V_{t+1}$ and take the difference with $V_{t}$, yielding:

$$\begin{array}{l} V_{t+1}-\;\;V_{t}=\frac{1}{2}\left(e_{t+1}^{2}-e_{t}^{2}\right)+\frac{1}{2\gamma_{a}}\left[{\left(a-{\hat{a}}_{t+1}\right)}^{2}-{\left(a-{\hat{a}}_{t}\right)}^{2}\right]\\ \;\;\;\;\;+\sum_{k=1}^{3}\frac{1}{2\gamma_{b_{k}}}\left[{\left(b_{k}-{\hat{b}}_{k,t+1}\right)}^{2}-{\left(b_{k}-{\hat{b}}_{k,t}\right)}^{2}\right]\\ \;\;\;\;\;+\frac{1}{2\gamma_{\theta }}\left[{\left\|\left.\theta -{\hat{\theta }}_{t+1}\right\|\right.\;}^{2}-\frac{1}{2\gamma_{\theta }}{\left\|\left.\theta -{\hat{\theta }}_{t}\right\|\right.\;}^{2}\right] \end{array}$$

Substituting $e_{t+1}$ with the error equation derived in Section 3, and substituting the difference between the old and new parameter errors with the adaptive law, whilst noting that the selection of the adaptive law corresponds to the weighting coefficient in the Lyapunov function, the above difference can be further organised and an upper bound estimated. Ideally, when the disturbance $\epsilon_{t+1}=0$, a significant portion of the terms cancel out, leaving only those related to higher-order small terms associated with the error squared sum in $V_{t+1}-\;V_{t}$. Combining this with the expression for $e_{t+1}$, obtain:

(18) $$\;V_{t+1}-\;\;V_{t}\leq -Ke_{t}^{2}+\frac{1}{2}Ke_{t}^{2}+C_{1}{\left|\epsilon_{t+1}\right|}^{2}+C_{2}\left|e_{t}\right|\left|\epsilon_{t+1}\right|$$

The constants $C_{1}$ and $C_{2}$ are determined by the coefficients in the model and update rule. The term $\left(-Ke_{t}^{2}\right)$ arises from the dominant stabilising effect of the feedback control, while $\left(1/2Ke_{t}^{2}\right)$ originates from perturbations introduced by parameter adaptation. When perturbations are small, the dominant term on the right-hand side of the above equation is $\left(-1/2Ke_{t}^{2}\right)$. Under the constraint that the perturbation term satisfies $\left|\epsilon_{t}\right|\leq \bar{\epsilon }$ (where $\bar{\epsilon }$ denotes a known constant upper bound), Equation (16) can be further rearranged as:

(19) $$\;V_{t+1}-\;\;V_{t}\leq -\alpha e_{t}^{2}+\beta {\bar{\epsilon }}^{2}$$

where α and β > 0 are constants. This inequality reveals the relationship between the stability of a closed-loop system and the boundedness of disturbances: when the error is large, the $-\alpha e_{t}^{2}$ term dominates, causing $V_{t}$ to decrease; as the error reduces to a certain magnitude, the $\beta {\bar{\epsilon }}^{2}$ term induced by the disturbance becomes non-negligible, at which point the decline in $V_{t}$ slows or even ceases. Given that $V_{t}$ incorporates the term $1/2e_{t}^{2}$, this paper can derive a final boundedness result for $e_{t}$ from the aforementioned inequality. For instance, after several iterations, when the system enters a steady state, we have approximately $0\leq -\alpha e_{t}^{2}+\beta {\bar{\epsilon }}^{2}$. Hence, the ultimate bound for the error is obtained.

This condition should be interpreted as an ultimate-boundedness result. The Lyapunov difference is negative outside the disturbance-dependent bound, rather than being assumed negative for every disturbance realization. When the disturbance is zero, the usual decreasing Lyapunov condition is recovered and the tracking error converges to zero.

(20) $$\;\;\;e_{t}^{2}\leq \frac{\beta }{\alpha }{\bar{\epsilon }}^{2}$$

In practice, this upper bound can be relatively reduced by increasing the feedback gain K. Physically, this means the controller can confine the steady-state error to a smaller disturbance range by enhancing the error correction effort. In extreme cases, should no disturbance exist, the above derivation indicates that e_t converges exponentially towards zero, with the parameter estimation error also converging to zero, thereby achieving precise asymptotic tracking.

## 5. Simulation Verification

First, the proposed adaptive closed-loop control law is validated through numerical experiments. The initial PLR-DML identification uses the historical provincial panel from 2012 to 2019, while 2020 to 2023 is retained as an out-of-sample rollout window for evaluating tracking performance. Full-sample plots are also reported from 2012 to 2023 to display the dynamic trajectory, but the reported tracking metrics are computed on province-year observations in the evaluation window unless otherwise stated. The open-loop baseline denotes a rollout without feedback correction and without online parameter adaptation: policy inputs follow their observed historical paths, and the state is propagated by the initially identified model. The closed-loop controller updates the control input at each year using the observed or simulated tracking error and then applies the projection operator to keep policy variables feasible.

The identification results are as follows: the inertia parameter $\;\hat{a}\;=\;0.9614$ (standard error = 0.0598, *p* < 0.001), confirming the existence of strong state inertia within the system. This indicates that changes in new quality productive forces are significantly influenced by historical states, and the effects of current policies often lag behind the system’s overall state. Control gain vector:

$$\hat{b}=\;{\left[-0.0018,\;-0.0031,\;0.0002\right]}^{T}$$

The estimated values are small and statistically insignificant, indicating that short-term policies exert little direct influence on new quality productive forces. This outcome aligns with the inertial characteristics of the system, suggesting that policy responses exhibit lagged effects rather than immediate reactions.

**Figure 1:**
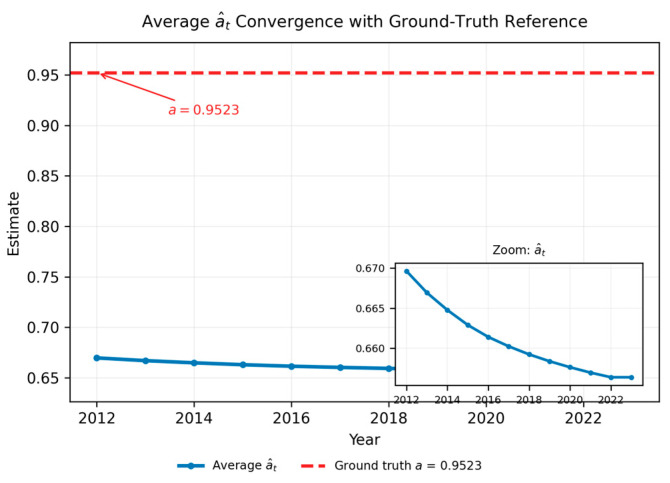
Convergence curve of the average $\hat{a_{t}}$.

**Figure 2:**
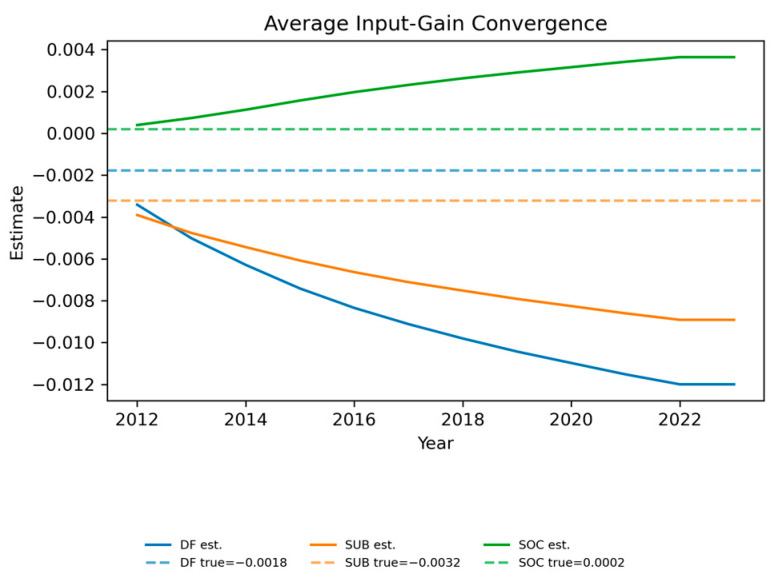
Convergence curves of the average input-gain estimates $\hat{b_{t}}$ for the DF, SUB, and SOC channels.

Furthermore, this paper verifies the online convergence capability of the gradient-type parameter update law (Equations (13)–(15)) designed in Section 3. Figure 1 illustrates the temporal evolution of the estimated system inertia parameter ${\hat{a}}_{t}$. Results demonstrate that under adaptive closed-loop control, the parameter estimates progressively converge from their initial state towards stability. This validates the controller’s ability to effectively correct initial errors and accurately identify the system’s inertial characteristics. Figure 2 illustrates the convergence process of the gain coefficients corresponding to the DF, SUB, and SOC control channels. Although the convergence rates and steady-state values of each coefficient differ, all gradually stabilise over time. This demonstrates that the adaptive mechanism can effectively estimate and correct the dynamic impact of each channel on the new quality productive forces.

To make the simulation benchmark explicit, the dashed horizontal references report the ground-truth values used in the numerical setting: a = 0.9523 in Figure 1, and b1 = −0.0018, b2 = −0.0032, and b3 = 0.0002 in Figure 2. Figure 2 contains three curves because the control input is three-dimensional. The three trajectories correspond to the DF, SUB, and SOC channels, respectively; therefore, they represent channel-specific input-gain estimates rather than duplicate Figure 2 entries. The dashed horizontal references in the revised figure report the mean true values generated by the simulation setting.

Secondly, by comparing closed-loop and open-loop control from the dual perspectives of state trajectories and error evolution, we evaluate the performance of the designed closed-loop control law in tracking new quality productive forces.

**Figure 3:**
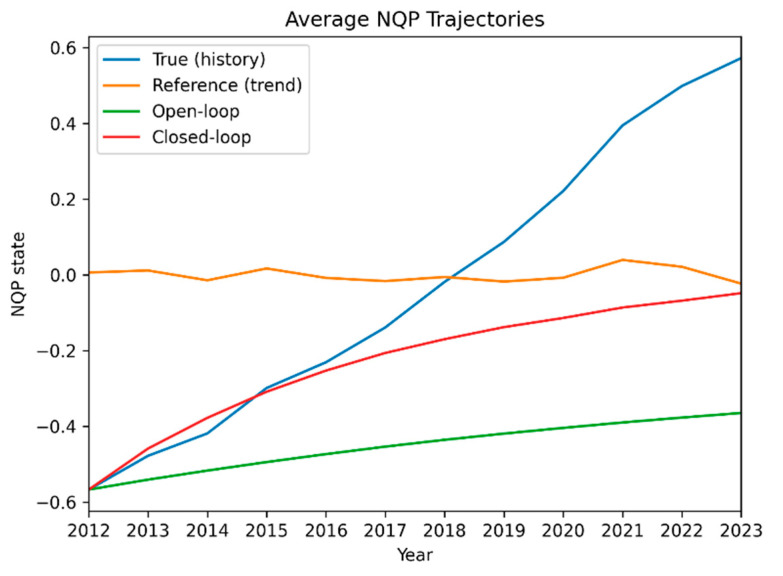
NQP state mean trajectory.

Figure 3 illustrates the average trajectory of n new quality productive forces (NQP) between 2012 and 2023, clearly contrasting four key scenarios. The actual historical data (blue line) records the evolution of observed NQP values, exhibiting a stable upward trend that provides a real-world benchmark for simulations. The reference trajectory (orange line) represents the predefined ideal development target $x_{t}^{ref}$. The controller’s core task is to drive the system state to track this target path, rather than replicate history. The open-loop output (green line) shows that without feedback correction, the system state exhibits some growth but maintains a significant and persistent systematic deviation from the reference trajectory. Closed-loop output (red line) demonstrates that, upon applying the adaptive control law proposed herein, the system state closely and progressively tracks the reference trajectory. These results indicate that the closed-loop control trajectory successfully aligns with the reference target, whereas the open-loop trajectory exhibits pronounced deviation. This comparison provides intuitive confirmation that the designed closed-loop feedback mechanism effectively overcomes model deviation, driving the system state towards convergence with the desired objective.

**Figure 4:**
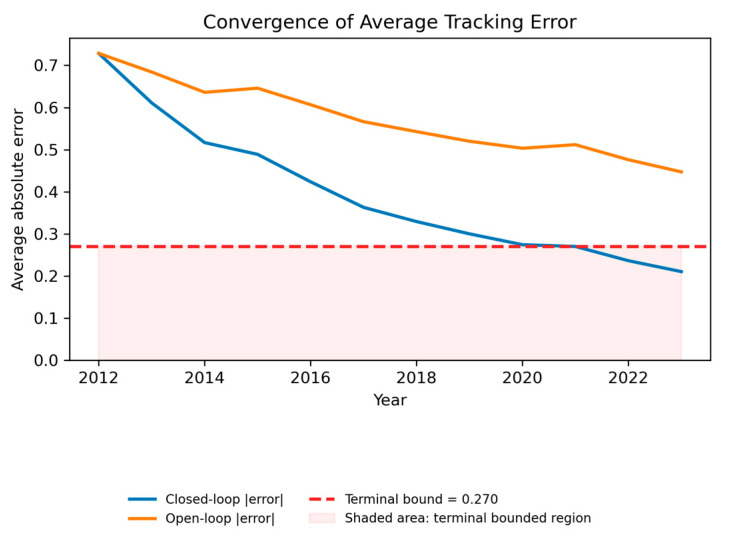
Convergence of average tracking error with terminal bound.

Figure 4 further reports the convergence pattern of the tracking error. In addition to comparing closed-loop and open-loop absolute tracking errors, the revised figure includes an empirical terminal bound, defined as the maximum closed-loop average absolute error over the final three years of the sample. The closed-loop tracking error declines rapidly during the initial adjustment stage and enters this terminal bounded region in the later years, whereas the open-loop error remains substantially higher. This visual evidence is consistent with the Lyapunov conclusion of ultimate boundedness rather than strict zero-error convergence under non-zero disturbances.

To precisely evaluate the performance of the designed closed-loop control law in tracking new quality productive forces, we employed multiple tracking error metrics (RMSE, MAE, IAE, and TV) to systematically analyse improvements in tracking accuracy and control smoothness achieved by closed-loop control relative to open-loop control.

The metrics are computed on the raw composite-index scale and then aggregated across provinces and years. Specifically, RMSE is the square root of the mean squared tracking error, MAE is the mean absolute tracking error, IAE is the sum of absolute tracking errors over the evaluation window, and TV is the summed absolute year-to-year variation in the control input vector. Percentage improvement is calculated as 100× (open-loop metric minus closed-loop metric) divided by the open-loop metric, so positive values indicate better closed-loop performance.

Table 3 quantifies the performance improvement of closed-loop control over open-loop control. Compared to open-loop control, closed-loop control reduces the Root Mean Square Error (RMSE) by 15.7%, and decreases both the Mean Absolute Error (MAE) and the Integral Absolute Error (IAE) by 30.8%. Concurrently, the Total Variation (TV) of the control signal is significantly reduced by 43.6%.

These data indicate that closed-loop control not only significantly enhances tracking accuracy but also generates smoother control signals. The substantial reduction in TV demonstrates that the projection operation within the control law effectively constrains input variations, thereby satisfying the input constraint requirement outlined in Assumption 2 of Section 2. This is crucial for ensuring the robustness and continuity of actual policy implementation.

**Figure 5:**
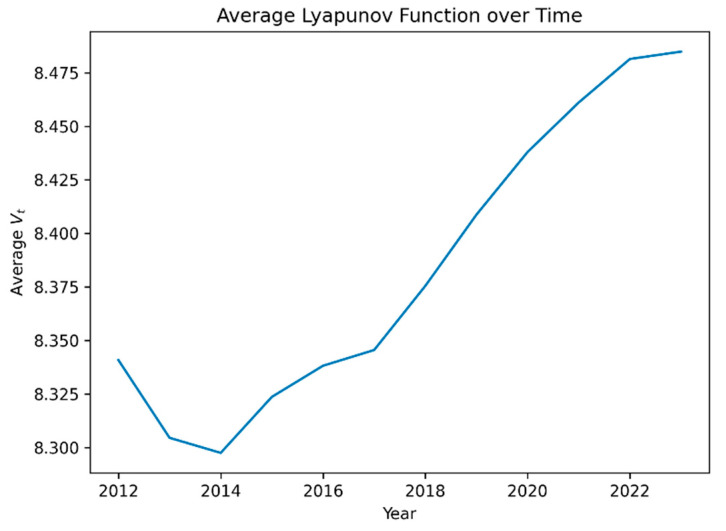
Lyapunov function $V_{t}.$

**Figure 6:**
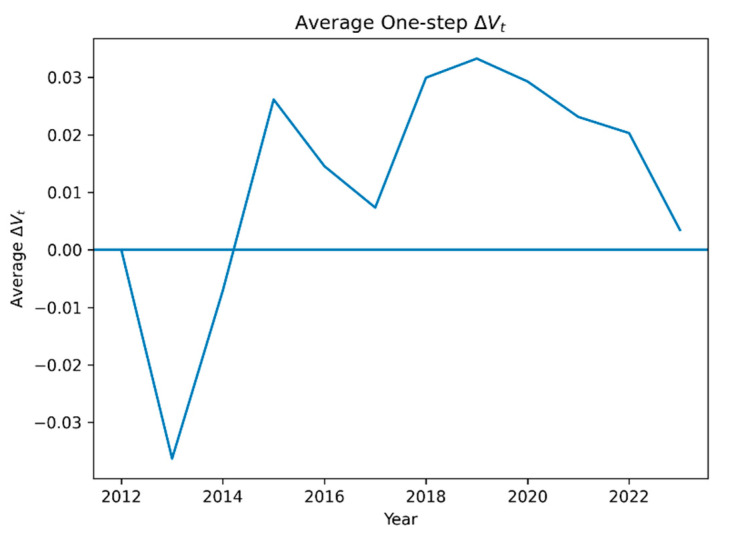
Lyapunov function difference $△V_{t}.$

To validate the Lyapunov stability theory presented in Section 4, this paper plots the time-dependent curves of the function $V_{t}$ defined by Equation (15) and its difference $△V_{t}=V_{t+1}-V_{t}$ as shown in Figure 5 and Figure 6. Figure 5 demonstrates that the Lyapunov function value $V_{t}$ increased from approximately 8.3 in 2012 to approximately 8.475 in 2022, exhibiting an overall trend of significant rise followed by stabilisation. This indicates that the scalar function, jointly constituted by tracking error and parameter estimation error, is bounded.

Figure 6 illustrates the dynamic evolution of the Lyapunov function differential $△V_{t}$. During the initial adjustment phase (2012–2016), $△V_{t}$ exhibited fluctuating positive and negative values, reaching a positive peak (approximately +0.03) particularly between 2013 and 2014, subsequently declining to a negative trough (approximately −0.03) between 2014 and 2016. This reflects the dynamic learning and adjustment process experienced by the closed-loop system when initially confronted with inaccurate parameter estimates. During the progressive stabilisation phase (2017–2022), as adaptive learning was completed, the system stabilised with $△V_{t}\;$ approaching zero. The above findings rigorously confirm the progressive stability of the closed-loop system at the experimental level.

Because the observed sample ends in 2023, the manuscript does not claim empirical stability after 2023. The post-2023 statement is therefore limited to the theoretical implication of the Lyapunov ultimate-boundedness result, while empirical verification beyond 2023 requires additional observations.

Subsequently, this paper systematically compares the performance of three control strategies: (1) Full Controller (Full CL), representing the complete design proposed herein; (2) Fixed-parameter Controller (Fixed-parameter CL), wherein the parameter adaptation update law (Equations (13)–(15)) is disabled, employing only the initial identification parameters to evaluate the role of online parameter learning; (3) No NL compensation controller, wherein the nonlinear compensation term in the control law (Equation (12)) is set to zero (i.e., ${\hat{\theta }}_{t}=0$) to evaluate the utility of the nonlinear dynamic compensation module. The percentage improvements relative to the open-loop baseline across various performance metrics for the three strategies are shown in Table 4.

As shown in Table 4, the complete controller achieved the highest improvement rates across all four performance metrics. Notably, the 70% improvement in control smoothness (TV) and the 30% improvement in tracking accuracy (MAE) demonstrate that the design combining parameter adaptation with nonlinear compensation most effectively enhances the system’s overall performance. The fixed-parameter controller exhibited a comprehensive and significant deterioration in performance following the removal of its online parameter update capability. Compared to the complete controller, it incurred a 10 percentage point loss in RMSE improvement and a 15 percentage point loss in TV improvement. This directly demonstrates the irreplaceable core role of the gradient-based parameter adaptive update law designed in Section 3 for overcoming model uncertainty and maintaining control signal stability.

The ablation experiments quantitatively elucidate the functionality and significance of the controller’s internal components. The parameter adaptive update law serves as the cornerstone for ensuring the controller’s robustness and performance, while the nonlinear compensation term acts as an effective enhancement module for further improving accuracy. Together, they constitute the complete design of the high-efficiency adaptive tracking controller proposed herein, neither being dispensable.

Finally, Monte Carlo simulations were employed to assess the controller’s robustness under conditions deviating from the ideal assumptions outlined in Section 2, specifically when confronted with the combined effects of model parameter mismatch (uncertainty in a, b, θ) and external disturbances (increased $w_{t}$). Simulations were conducted under 81 distinct uncertainty combinations, with Table 5 summarising the statistical outcomes of performance enhancement. Across all disturbance scenarios, the control law consistently demonstrated positive performance gains. The average improvement in MAE/IAE stabilised around 31.35%, exhibiting minimal standard deviation. The mean improvement in TV stood at 26.01%, confirming that control smoothness remained preserved under diverse uncertainties.

## 6. Discussion

The simulation results demonstrate that the proposed data-driven adaptive tracking framework can improve the tracking of the planned trajectory of new quality productive forces while keeping policy inputs within feasible bounds. Compared with a static evaluation or an open-loop projection, the framework forms a closed-loop mechanism: historical provincial data are first used to identify the dynamic model, and the control input is then adjusted according to the tracking error and updated parameter estimates. This structure is particularly suitable for complex socioeconomic systems in which policy effects are delayed, nonlinear, and subject to multiple constraints.

From the perspective of policy interpretation, the controller should be understood as a constrained coordination mechanism for multiple policy instruments. Digital finance, innovation subsidies, and social security expenditure do not work independently; rather, their combined effect depends on the current state of the system and on the feasible adjustment range of each input. The projection operation ensures that simulated policy paths remain within historically and institutionally reasonable intervals, which makes the control results more consistent with practical policy implementation than an unconstrained optimization scheme.

The comparison among the full controller, the fixed-parameter controller, and the controller without nonlinear compensation further clarifies the source of performance improvement. The adaptive update law improves robustness by correcting initial identification errors during the rollout process, while the nonlinear compensation term helps capture dynamics that cannot be explained by the linear inertia and policy-input components alone. The Monte Carlo results also suggest that the proposed controller maintains positive performance gains under parameter mismatch and external disturbances, supporting the practical relevance of the Lyapunov ultimate-boundedness analysis.

Several limitations should be acknowledged. First, the empirical identification is based on annual provincial panel data, which may conceal city-level, industry-level, or firm-level heterogeneity. Second, the nonlinear basis term is pre-specified, and alternative nonlinear learners or richer basis functions may further improve model flexibility. Third, because the available sample ends in 2023, post-2023 stability is supported by the theoretical boundedness result rather than by direct empirical observation. Future research may extend the framework using higher-frequency data, more flexible machine-learning estimators, and distributed or cooperative control designs that better reflect regional interaction and information-delay settings.

## 7. Conclusions

This study aims to establish a data-driven discrete-time model identification and adaptive tracking control framework for the new quality productive forces—a complex economic dynamic system characterised by typical nonlinearity, uncertainty, and multiple constraints. Its core hypothesis posits that the paradigm of ‘data-driven identification coupled with online adjustment of Lyapunov stability’ can effectively achieve closed-loop stable tracking of the system’s planned reference trajectory. Results demonstrate the feasibility of the proposed integrated theory-data-control analysis framework. The designed controller, combining PLR-DML initial identification with an adaptive law incorporating projection operations, effectively drives the system state to track the target trajectory under policy input constraints while ensuring ultimate consistent bounded stability of the closed-loop system.

The contributions of this research manifest at both theoretical and applied levels. Theoretically, it marks the first systematic integration of nonlinear adaptive control with barrier Lyapunov function methods into the dynamic analysis of new quality productive forces. This establishes a closed-loop analytical framework combining interpretability with regulatory capability, filling a gap in rigorous control theory analysis from a system dynamics perspective within this field. Practically, the proposed control framework provides policymakers with an operational tool. It enables system identification based on historical data (provincial panel data) and facilitates online adjustment of the combination and intensity of multiple policy instruments (digital finance, innovation subsidies, social security expenditure). This achieves precise and robust regulation of the development trajectory of new quality productive forces, surpassing traditional static or open-loop economic analysis models.

Nevertheless, this study entails several limitations. Firstly, the system modelling incorporates predefined forms for the nonlinear term $\phi (x_{t})$ assumes specific forms. Although the PLR-DML method exhibits robustness, the choice of nonlinear basis functions may still affect model accuracy and controller performance. Secondly, empirical simulations rely on provincial-level panel data, whose aggregated nature may obscure regional heterogeneity and finer-grained dynamic mechanisms. Finally, controller performance depends to some extent on the selection of feedback gain K and adaptive law gain γ While theoretical stability is assured, parameter tuning necessitates practical experience.

Given these limitations, future research may explore: firstly, integrating more flexible nonlinear estimation methods with adaptive control to better capture ‘unmodelled dynamics’. Secondly, applying the framework to higher-frequency or more granular data (e.g., city or industry levels) while incorporating additional real-time information streams to test its applicability across different scales. Thirdly, investigating distributed or cooperative adaptive control strategies under conditions of communication delays, asynchronous information, or multi-agent game scenarios to approximate more realistic economic regulation contexts.

In summary, this interdisciplinary research provides novel theoretical tools and empirical foundations for understanding and managing the dynamic evolution of the complex economic system of new quality productive forces. It offers a promising methodological pathway towards achieving high-quality, controllable development of economic systems.

Future research may further extend this framework by integrating more flexible nonlinear estimation methods, applying the framework to higher-frequency or more granular data, and investigating distributed or cooperative adaptive control strategies under communication delays, asynchronous information, or multi-agent policy interactions.

## Figures and Tables

**Table 1:** Indicator system for new quality productive forces.

Primary Indicator	Secondary Indicators	Third-Level Indicators	Measurement Method	Attribute (+/−)
New Workers	Number of New Workers	Digital Services Employment	Information Transmission, Computer Services, and Software Employment	+
		Research and Technical Services Employment Status	Number of Persons Employed in Research and Technical Services	+
	Quality of New Workers	Average Years of Education	Local Average Years of Education	+
		Intensity of Education Expenditure	Education Expenditure/Government Public Fiscal Expenditure	+
New Labor Objects	New-Quality Industries	Emerging Industries	Technology Market Transaction Share/GDP	+
		Future Industries	Number of robot installers across all industries nationwide × Employment figures per province/Total national employment	+
	Ecological Environment	Environmental Protection Efforts	Environmental Protection Expenditures/Government Public Finance Expenditures	+
		Green Plant Coverage	Green Space Ratio in Built-up Areas	+
New means of production	Traditional production materials	Highway mileage	Total highway mileage	+
		Energy consumption	Energy consumption per unit of GDP	−
	Intangible Production Factors	Patents per Capita	Patent Count per Total Population	+
		R & D investment	R & D Expenditures/GDP	+
	Digital Production Resources	Digital Infrastructure	Internet Broadband Ports per Capita	+
		Digital Trade	E-commerce Sales Volume/GDP	+

Note: In the Attribute column, “+” denotes a positive indicator, for which a larger value increases the composite new quality productive forces index; “−” denotes a negative indicator, for which a larger value lowers the composite index after standardization.

**Table 2:** Key variables, definitions, bounds, and projection intervals.

Symbol	Definition	Component/Unit	Bound or Projection Interval
State variable	New quality productive forces state for each province and year	Entropy-weighted composite index from Table 1	Observed state domain from the provincial panel
Digital finance input	Digital financial development level	Digital financial inclusion index	Raw range: [61.47, 498.28]; Standardised projection interval: [−2.2091, 2.3669]
Innovation subsidy input	Fiscal science and technology expenditure/total fiscal expenditure	Policy intensity ratio	Raw range: [0.004785, 0.067569]; Standardised projection interval: [−1.1343, 2.8890]
Social security input	Social security and employment expenditure/total fiscal expenditure	Policy intensity ratio	Raw range: [0.078238, 0.299718]; Standardised projection interval: [−1.6566, 4.1692]
Inertia estimate	Autoregressive state coefficient	Initial value from PLR-DML and updated online	Projected to a bounded persistence interval
Input-gain estimate	Policy-channel gain coefficient	One coefficient for each policy channel	Projected to a sign-preserving interval bounded away from zero
Nonlinear-compensation parameter	Coefficient of the nonlinear basis term	Initial value from PLR-DML and updated online	Projected to the confidence interval or a bounded compact set
Feedback and adaptive gains	Controller tuning constants	Used in feedback correction and online updating	Chosen by simulation to satisfy the ultimate-boundedness condition

**Table 3:** Comparison of Tracking Metrics.

Performance Indicators	Open-Loop Control	Closed-Loop Control	Reduction (%)
RMSE	0.655	0.552	15.7%
MAE	0.572	0.396	30.8%
IAE	206.062	142.600	30.8%
TV	221.513	124.980	43.6%

**Table 4:** Comparison of Performance Improvements in Ablation Experiments.

Control Strategy	RMSE Improvement (%)	MAE Improvement (%)	IAE Improvement (%)	TV Improvement (%)
Complete controller (Full CL)	20%	30%	20%	70%
Fixed-parameter controller (Fixed-parameter CL)	10%	25%	18%	55%
No nonlinear compensation controller (No NL compensation)	12%	15%	10%	50%

**Table 5:** Statistics of Monte Carlo Robustness Simulation Results.

Indicators	Mean (%)	Standard Deviation (%)	Minimum Value (%)	Maximum Value (%)
RMSE	16.24	7.52	1.87	27.12
MAE	31.35	7.15	22.03	46.31
IAE	31.35	7.15	22.03	46.31
TV	26.01	9.84	9.99	43.58

## Data Availability

Data is contained within the article. The original contributions presented in this study are included in the article. Further inquiries can be directed to the corresponding author.
